# Association between perioperative allogenic blood transfusion and risk of fracture related infection and nonunion in operatively treated tibial shaft fractures

**DOI:** 10.1007/s00590-026-04759-1

**Published:** 2026-04-29

**Authors:** Jeremy M. Adelstein, Tyler J. Moon, Lucas R. Haase, Victoria J. Nedder, Anna M. Swetz, Logan M. Good, George Ochenjele, Robert J. Wetzel, John K. Sontich, Nicholas M. Romeo, Joshua K. Napora

**Affiliations:** 1 Department of Orthopaedic Surgery, University Hospitals Cleveland Medical Center, Cleveland, United States; 2 School of Medicine, Case Western Reserve University, Cleveland, United States; 3 Department of Orthopaedic Surgery, MetroHealth Medical Center, Cleveland, United States

**Keywords:** Nonunion, Fracture related infection, Allogenic blood transfusion, Tibial shaft fracture

## Abstract

**Introduction:**

Recent orthopaedic trauma literature has linked blood transfusions with poor outcomes due to proposed deleterious effects on healing. The aim of this study was to determine the association of perioperative allogenic blood transfusion after tibial shaft fixation with nonunion and fracture related infection (FRI).

**Methods:**

A retrospective cohort of patients at two level 1 trauma centers over a 12-year period who sustained extra-articular tibial shaft fractures treated with intermedullary nailing (IMN) or open reduction internal fixation (ORIF). A minimum follow-up of six months was required unless there was presence of clinical and radiographic union prior to six months. Peri-operative allogenic red blood cell transfusion was the primary independent variable. Outcomes included fracture-related infection (FRI) and nonunion. Sub-analyses were completed in patients with open and closed fractures separately.

**Results:**

550 tibia fractures met inclusion criteria. Mean age was 45 years. 333 (61%) patients were male. 247 (45%) patients were current tobacco users. 335 (59%) patients sustained a high energy fracture mechanism. 71 fractures (13%) were classified as AO/OTA 42-C and 230 fractures (42%) were open. Mean follow up was 18.2 ± 17 months. 138 patients (25%) received a peri-operative blood transfusion. 54 patients (10%) were diagnosed with FRI and 86 patients (16%) were diagnosed with nonunion. Multivariate regression analysis demonstrated no independent association between transfusion and FRI for the whole cohort (OR 1.6; 95% CI 0.9–2.9; *p* = 0.1) or in patients with open fracture (N = 230, 19% transfusion versus 14% no transfusion, *p* = 0.3). Multivariate regression analysis demonstrated no independent association between transfusion and nonunion for the whole cohort (OR 1.8; 95% CI 0.9–3.3; *p* = 0.1) but did demonstrate an independent association for patients with open fractures (N = 230, OR 2.0; 95% CI 1.1–3.7; *p* = 0.03).

**Conclusions:**

This multicenter study demonstrated that nonunion, but not FRI, may be associated with peri-operative red blood cell transfusion in open tibial shaft fractures. Surgeons may consider indications for transfusion after open tibia fracture fixation to minimize risk of poor outcomes.

**Level of Evidence:**

Level III Prognostic.

**Supplementary Information:**

The online version contains supplementary material available at 10.1007/s00590-026-04759-1.

## Introduction

Complication rates after operative treatment of tibial shaft fractures are high, with reported nonunion rates between 7–13% [1–4] and fracture-related infection (FRI) rates between 1–4% for all fractures [5, 6] and up to 30% for open fractures [7, 8]. Multiple risk factors for complications after operative treatment of tibial shaft fractures have been identified, including male sex, older age, diabetes, tobacco use, higher energy mechanisms, open fracture, and fracture malreduction [2, 5, 9, 10]. Recent studies have also identified allogenic blood transfusion as a risk factor for poor outcomes in orthopedic trauma [11, 12], spine [13, 14], and arthroplasty [15, 16], as well as in surgical procedures outside of the orthopedic field [17, 18]. Allogenic blood transfusion generates an immunomodulation cascade that increases both pro-inflammatory and immunosuppressive effects and can have deleterious effects on healing and recovery after trauma and surgery [19–22]. No study to date has investigated the impacts of perioperative allogenic blood transfusion on outcomes after operative treatment of tibial shaft fractures. It was hypothesized that perioperative allogenic blood transfusion would be associated with increased rates of FRI and nonunion after surgical treatment of tibial shaft fractures.

## Methods

Following institutional review board approval, all patients treated operatively for tibial shaft fractures at two level 1 trauma centers between January 1, 2010, and December 31, 2021, were retrospectively reviewed. A billing database search by Current Procedural Terminology codes was completed to include patients who underwent open reduction and internal fixation (ORIF) (CPT 27758) or intramedullary nailing (IMN) (CPT 27759) for tibial shaft fracture. Direct intra-articular fractures were excluded. A minimum follow-up time was set at 6 months or time to clinical and radiographic union. A follow-up time of 6 months was determined to include patients who had demonstrated fracture union at 6 months but did not sustain more long-term follow-up.

Patient charts were reviewed using electronic medical records. Patient specific characteristics including age, sex, obesity, tobacco use, and medical comorbidities were recorded as well as injury characteristics including fracture mechanism, open injury, and fracture pattern based on the AO/OTA classification. Low energy fracture mechanisms included falls from standing height or low energy ballistic injuries, while high energy mechanisms included motor vehicle accidents, bicycle accidents, falls from greater than standing height, pedestrians struck by motor vehicles, and high energy ballistic injuries. Peri-operative allogenic red blood cell transfusion was recorded as a binary variable. Peri-operative transfusion was defined as any transfusion on postoperative day 0 (POD0), including preoperatively, until postoperative day 5 (POD5). Transfusion of red blood cells was ordered at the discretion of the primary hospital team (orthopedic surgery, general trauma surgery, or hospitalist medicine); standard transfusion threshold at both study institutions is hemoglobin < 7.0 g/dL or hemoglobin > 7.0 g/dL with symptoms of anemia. Transfusion of fresh frozen plasma, platelets, and other blood products were not recorded. Postoperative outcomes including superficial infection, deep infection, and nonunion were recorded. Superficial infection was defined as delayed wound healing requiring local wound care or oral antibiotics without return to the operating room for debridement. Deep infection was defined as an infection at the site of initial surgery requiring a return to the operating room for debridement. Nonunion was determined by each treating surgeon based on the need for surgical intervention to induce fracture healing. This was felt to be a more reliable identifier of nonunion considering the fellowship-trained trauma faculty often do not abide by strict standardized diagnostic criteria for nonunion and often implement some clinical gestalt.

All data were stored electronically and analyzed using the Statistical Package for Social Sciences (Version 29; IBM SPSS, Armonk, New York). Patients were grouped by transfusion status. Independent two-sample *t*-tests were used to compare continuous variables and Chi-squared tests were used to compare categorical variables. In multivariable analysis, effects of perioperative allogenic blood transfusion on FRI and nonunion were assessed by a logistic regression model to adjust for the potential confounding effects of other variables. Confounding variables were included in regression analysis if their *P* value was less than 0.05 on univariate analysis for the outcome variables. Variables with *P* values less than 0.05 with respect to transfusion status were also included in regression analysis. Sub-analysis was completed similarly for the cohorts of patients with just open or closed fractures. *P* values less than 0.05 were considered significant for all analyses.

## Results

893 patients were initially identified to have undergone operative treatment of tibial shaft fractures during the study period. 235 patients were excluded for < 6 months follow-up without documented union. 108 patients were excluded for intra-articular extension of their primary fracture. 550 patients remained in the final cohort for analysis.

Demographics and injury information analyzed with respect to transfusion status are listed in Table 1. The mean age was 45 years. 333 patients (61%) were male. 247 patients (45%) were current tobacco users. 335 (59%) patients sustained a high energy fracture mechanism, and 230 patients (42%) sustained an open fracture. 267 fractures (48%) were classified as simple (AO/OTA 42A), 212 fractures (39%) were classified as wedge (AO/OTA 42B), and 71 fractures (13%) were classified as complex (AO/OTA 42C). Mean follow up was 18.2 ± 17 months. 138 patients (25%) received a peri-operative blood transfusion. Patients were more likely to require a transfusion if they had a high energy fracture mechanism (*p* < 0.001), open fracture (*p* < 0.001), and more severe AO/OTA fracture classification (*p* < 0.001).

**Table 1:** Demographic and injury information for patient population who did and did not have an allogenic blood transfusion during initial hospital stay

	Total (N = 550)	No Transfusion (N = 412)	Transfusion (N = 138)	*p* value
Age (years)	45 ± 17	44 ± 16	46 ± 17	0.2^a^
Male Sex	333 (61%)	252 (61%)	81 (59%)	0.6^b^
Obese (BMI > 30 kg/m^2^)	190 (35%)	142 (35%)	48 (35%)	0.9^b^
Current tobacco use	247 (45%)	186 (45%)	61 (44%)	0.8^b^
Diabetes mellitus	106 (19%)	74 (18%)	32 (23%)	0.2^b^
High energy mechanism	335 (61%)	224 (54%)	111 (80%)	** < 0.001**^**b**^
Open fracture	230 (42%)	146 (35%)	84 (61%)	** < 0.001**^**b**^
GA Type I	56 (10%)	43 (10%)	13 (9%)	
GA Type II	66 (12%)	52 (13%)	14 (10%)	
GA Type IIIa	84 (15%)	48 (12%)	36 (26%)	
GA Type IIIb	15 (3%)	2 (1%)	13 (9%)	
GA Type IIIc	9 (2%)	1 (1%)	8 (6%)	
Fracture pattern				** < 0.001**^**b**^
Simple (AO/OTA 42A)	267 (48%)	221 (54%)	46 (33%)	
Wedge (AO/OTA 42B)	212 (39%)	147 (36%)	65 (47%)	
Complex (AO/OTA 42C)	71 (13%)	44 (11%)	27 (19%)	
Definitive fixation method				0.1^b^
Intramedullary nail (IMN)	461 (84%)	344 (84%)	117 (85%)	
Open reduction internal fixation (ORIF)	56 (10%)	47 (11%)	9 (7%)	
IMN + open plating	33 (6%)	21 (5%)	12 (9%)	
Fracture-related infection	54 (10%)	32 (8%)	22 (16%)	**0.01**^**b**^
Superficial infection	72 (13%)	53 (13%)	19 (14%)	0.8^b^
Nonunion	86 (16%)	50 (12%)	36 (26%)	** < 0.001**^**b**^

Values reported as Mean ± Standard Deviation for continuous variables and N (%) for categorical variables.

All P-values considered significant if < 0.050 and are highlighted in bold.

a = Calculated using Student’s T-Test; b = Calculated using Pearson’s Chi-Squared Test.

BMI = body mass index; GA = Gustilo-Anderson.

Table 2 demonstrates univariate results with respect to FRI. 54 patients (10%) were diagnosed with FRI. Patients with diabetes mellitus (*p* = 0.04), open fracture (*p* < 0.001), and transfusion (p = 0.005) were more likely to develop FRI. Multivariate binary logistic regression for FRI is demonstrated in Table 3. Open fracture (OR 2.6; 95% CI 1.4–4.9; *p* = 0.003) and diabetes mellitus (OR 2.0; 95% CI 1.1–3.9; *p* = 0.04) were independently associated with FRI, while transfusion status was not (OR 1.6; 95% CI 0.9–2.9; *p* = 0.1). Sub-analysis for FRI in patients with open fracture only (N = 230) did not demonstrate a univariate association between FRI and transfusion (19% transfusion versus 14% no transfusion, *p* = 0.3) (Supplemental Table 1). Similarly, in patients with closed fractures (N = 320) there was no univariate association between FRI and transfusion (11% transfusion versus 5% no transfusion, *p* = 0.1).

**Table 2:** Demographic and injury factors associated with fracture related infection (FRI). BMI = body mass index

	Total (N = 550)	No FRI (N = 496)	FRI (N = 54)	*p* value
Age (years)	45 ± 17	45 ± 17	45 ± 16	0.9^a^
Male Sex	333 (61%)	296 (60%)	37 (69%)	0.2^b^
Obese (BMI > 30 kg/m^2^)	190 (35%)	165 (33%)	25 (46%)	0.1^b^
Current tobacco use	247 (45%)	216 (44%)	31 (57%)	0.1^b^
Diabetes mellitus	106 (19%)	90 (18%)	16 (30%)	**0.04**^**b**^
High energy mechanism	335 (61%)	296 (60%)	39 (72%)	0.1^b^
Open fracture	230 (42%)	194 (39%)	36 (67%)	** < 0.001**^**b**^
GA Type I	56 (10%)	50 (10%)	6 (11%)	
GA Type II	66 (12%)	60 (12%)	6 (11%)	
GA Type IIIa	84 (15%)	69 (14%)	15 (28%)	
GA Type IIIb	15 (3%)	9 (2%)	6 (11%)	
GA Type IIIc	9 (2%)	6 (1%)	3 (6%)	
Fracture pattern				0.2^b^
Simple (AO/OTA 42A)	267 (48%)	246 (50%)	21 (39%)	
Wedge (AO/OTA 42B)	212 (39%)	189 (38%)	23 (43%)	
Complex (AO/OTA 42C)	71 (13%)	61 (12%)	10 (19%)	
Definitive fixation method				0.9^b^
Intramedullary nail (IMN)	461 (84%)	417 (84%)	44 (82%)	
Open reduction internal fixation (ORIF)	56 (10%)	50 (10%)	6 (11%)	
IMN + Open Plating	33 (6%)	29 (6%)	4 (7%)	
Transfusion	138 (25%)	116 (23%)	22 (41%)	**0.005**^**b**^

Values reported as Mean ± Standard Deviation for continuous variables and N (%) for categorical variables.

All P-values considered significant if < 0.050 and are highlighted in bold.

a = Calculated using Student’s T-Test; b = Calculated using Pearson’s Chi-Squared Test.

BMI = body mass index.

**Table 3:** Results of multivariate binary logistic regression for fracture-related infection (FRI) including transfusion and all possible confounding variables that associated with FRI or differed between transfusion groups

N = 550	β	S.E	*p* value	Exp(β) [95% CI]
Open fracture	1.0	0.3	**0.003**	2.6 [1.4, 4.9]
Diabetes mellitus	0.7	0.3	**0.04**	2.0 [1.1, 3.9]
Transfusion	0.5	0.3	0.1	1.6 [0.9, 2.9]
High energy mechanism	0.3	0.4	0.4	1.3 [0.7, 2.6]
AO/OTA classification	–	–	0.8	–
42A	Reference	Reference	Reference	Reference
42B	0.1	0.3	0.7	1.2 [0.6, 2.2]
42C	0.3	0.4	0.5	1.3 [0.6, 3.1]
Constant	− 3.3	0.4	** < 0.001**	0.1

All included variables had p < 0.05 on univariate analysis. Significant variables defined as p < 0.05 and are highlighted in bold.

Table 4 demonstrates univariate results with respect to nonunion. 86 patients (16%) were diagnosed with nonunion. Patients with male sex (*p* < 0.001), current tobacco use (*p* < 0.01), high energy fracture mechanism (*p* = 0.005), open fracture (*p* < 0.001), more severe AO/OTA fracture pattern (*p* < 0.001), and transfusion (*p* < 0.001) were more likely to develop nonunion. Multivariate binary logistic regression for nonunion is demonstrated in Table 5. Open fracture (OR 2.6; 95% CI 1.3–4.7; *p* = 0.005) was the only variable independently associated with nonunion; transfusion status was not independently associated with nonunion within the entire cohort (OR 1.8; 95% CI 0.9–3.3; *p* = 0.1).

**Table 4:** Demographic and injury factors associated with nonunion. BMI = body mass index

	Total (N = 550)	No Nonunion (N = 464)	Nonunion (N = 86)	*p *value
Age (years)	45 ± 17	44 ± 17	44 ± 15	0.9^a^
Male sex	333 (61%)	267 (58%)	66 (77%)	** < 0.001**^**b**^
Obese (BMI > 30 kg/m^2^)	190 (35%)	161 (35%)	29 (34%)	0.9^b^
Current tobacco use	247 (45%)	198 (43%)	49 (57%)	**0.01**^**b**^
Diabetes mellitus	106 (19%)	88 (19%)	18 (21%)	0.7^b^
High energy mechanism	335 (61%)	271 (58%)	64 (74%)	**0.005**^**b**^
Open fracture	230 (42%)	167 (36%)	63 (73%)	** < 0.001**^**b**^
GA Type I	56 (10%)	43 (9%)	13 (15%)	
GA Type II	66 (12%)	53 (11%)	13 (15%)	
GA Type IIIa	84 (15%)	60 (13%)	24 (28%)	
GA Type IIIb	15 (3%)	7 (2%)	8 (9%)	
GA Type IIIc	9 (2%)	4 (1%)	5 (6%)	
Fracture pattern				** < 0.001**^**b**^
Simple (AO/OTA 42A)	267 (48%)	245 (53%)	22 (26%)	
Wedge (AO/OTA 42B)	212 (39%)	166 (36%)	46 (54%)	
Complex (AO/OTA 42C)	71 (13%)	53 (11%)	18 (21%)	
Definitive fixation method				0.4^b^
Intramedullary nail (IMN)	461 (84%)	392 (84%)	69 (80%)	
Open reduction internal fixation (ORIF)	56 (10%)	44 (10%)	12 (14%)	
IMN + open plating	33 (6%)	28 (6%)	5 (6%)	
Transfusion	138 (25%)	102 (22%)	36 (42%)	** < 0.001**^**b**^

Values reported as Mean ± Standard Deviation for continuous variables and N (%) for categorical variables.

All P-values considered significant if < 0.050 and are highlighted in bold.

a = Calculated using Student’s T-Test; b = Calculated using Pearson’s Chi-Squared Test.

BMI = body mass index.

**Table 5:** Results of multivariate binary logistic regression for nonunion including transfusion and all possible confounding variables that associated with nonunion or differed between transfusion groups

N = 550	β	S.E	p-value	Exp(β) [95% CI]
Open fracture	0.9	0.3	**0.005**	2.5 [1.3, 4.7]
Transfusion	0.6	0.3	0.1	1.8 [0.9, 3.3]
Current tobacco use	0.4	0.3	0.2	1.5 [0.8, 2.7]
Male Sex	0.1	0.3	0.7	1.2 [0.6, 2.2]
AO/OTA classification	–	–	0.9	–
42A	Reference	Reference	Reference	Reference
42B	0.1	0.3	0.9	1.1 [0.6, 2.0]
42C	0.2	0.4	0.6	1.2 [0.5, 2.9]
High energy mechanism	0.1	0.4	0.9	1.1 [0.5, 2.1]
Constant	− 3.3	0.4	** < 0.001**	0.1

All included variables had *p* < 0.05 on univariate analysis. Significant variables defined as *p* < 0.05 and are highlighted in bold.

Sub-analysis for nonunion in patients with open fracture only (N = 230) did demonstrate a significant univariate association between nonunion and transfusion (19% transfusion versus 14% no transfusion, *p* = 0.03, Supplemental Table 2). Male sex (*p* = 0.04), current tobacco use (*p* = 0.03), and more severe AO/OTA fracture pattern (*p* = 0.01) were also associated with nonunion in the open fracture cohort on univariate analysis. Multivariate binary logistic regression for nonunion in the open fracture cohort including all variables with *p* < 0.05 in Supplemental Table 2 is demonstrated in Table 6. Transfusion status was independently associated with nonunion in patients with open fractures on multivariate analysis (OR 2.0; 95% CI 1.1–3.7; *p* = 0.03). AO/OTA 42B and 42C fracture classification was also independently associated with nonunion in this analysis, while male sex and current tobacco use were not. For patients with closed fractures (N = 320), there was no univariate association between nonunion and transfusion (11% transfusion versus 6% no transfusion, *p* = 0.2).

**Table 6:** Results of multivariate binary logistic regression with nonunion outcome variable for sub-analysis group of patients with open fracture

N = 230	β	S.E	p-value	Exp(β) [95% CI]
Transfusion	0.7	0.3	**0.03**	2.0 [1.1, 3.7]
Male sex	0.6	0.4	0.1	1.7 [0.8, 3.6]
AO/OTA classification	–	–	**0.04**	–
42A	Reference	Reference	Reference	Reference
42B	0.9	0.4	**0.02**	2.4 [1.1, 4.9]
42C	1.0	0.5	**0.04**	2.6 [1.1, 6.4]
Current tobacco use	0.5	0.3	0.1	1.6 [0.9, 3.0]
Constant	− 2.5	0.4	** < 0.001**	0.1

The model included transfusion and all possible confounding variables that are associated with nonunion. All included variables had *p* < 0.05 on univariate analysis. Significant variables defined as *p* < 0.05 and are highlighted in bold.

## Discussion

This multicenter retrospective cohort study is the first to investigate the effect of peri-operative allogenic red blood cell transfusion on outcomes after operative treatment of tibial shaft fractures. The present study demonstrated an overall nonunion rate of 13% and FRI rate of 10%, both of which are on the high end of those reported in prior literature, likely due to a high percentage of the cohort sustaining high energy and open injuries [1, 2, 6, 8]. On univariate analysis of the entire cohort, transfusion status was significantly associated with nonunion and FRI; these results were not maintained on multivariate analysis except for in the setting of open fracture and nonunion. Open fracture is cited as the most important risk factor for nonunion after tibial shaft fracture [3, 4, 23], consistent with the results of the present study. It is likely that in the overall cohort, the strong association between nonunion and FRI with open fracture overshadowed the effect of transfusion status. However, when investigating only patients with open fractures, transfusion status did associate with nonunion independent of other confounding risk factors including male sex, tobacco use, and more severe injury pattern. Results of this study did not demonstrate a significant association between transfusion and nonunion for closed fractures or between transfusion and FRI for either closed or open tibial shaft fractures on multivariate analysis. Importantly, this study did not evaluate quality of reduction and fixation which are strongly associated with risk of nonunion [24].

Relatively few studies have investigated the impact of allogenic perioperative blood transfusion on outcomes after orthopedic surgery. Several studies in the arthroplasty and spine literature have demonstrated increased infection and re-operation rates for elective procedures with increased rates of peri-operative blood transfusion after controlling for medical comorbidities [13–16]. No identified studies in spine literature investigated outcomes related to pseudoarthrosis which may be analogous to fracture nonunion.

The majority of studies that focus on the impact of peri-operative blood transfusion after orthopedic trauma have focused on infection and mortality after hip fracture treated with either arthroplasty or internal fixation and have demonstrated mixed results. One study demonstrated increased risk of in-hospital urinary tract infection associated with transfusion status [25]. A conflicting study did not identify increased infection rates [26], but neither study followed patients beyond the acute hospital stay. Johnston, et al., demonstrated no difference associated with transfusion status in mortality or infection rates at 1-year post-operatively [27]. Greenhalgh, et al., did demonstrate an increased rate of mortality at 1 year after blood transfusion in hip fracture patients [28], while Smeets, et al., did not [29]. Haase, et al., investigated outcomes associated with transfusion in patients treated surgically for distal femur fractures and identified an association between transfusion status and FRI but not nonunion [11]. Their findings are opposite of those found in the present study for the sub-cohort of open tibial fractures, likely due to differences in patient demographics, higher-energy injury profile, and baseline rates of nonunion and FRI between distal femur and tibial shaft fractures.

Given the potential association with negative outcomes in orthopedic trauma patients identified in prior literature and suggested in the present study, recent interest has been developed investigating optimal transfusion thresholds in orthopedic trauma patients. Mullis, et al., recently published a randomized controlled trial of 99 patients comparing transfusion thresholds of 7.0 g/dL and 5.5 g/dL in orthopedic trauma patients outside of the initial resuscitative period. They demonstrated a decreased infection rate without differences in anemia-related complications in the more conservative transfusion group at 30 days post-operatively with plans for separate report of 1-year follow-up [21]. The present study supports a conservative threshold to minimize peri-operative transfusion as it may be beneficial to minimize nonunion in open tibial shaft fractures as well as infection in other fractures based on prior studies.

This study has several limitations. First, the results of our study simply represent a potential association between nonunion and transfusion in open tibial shaft fractures and does not imply causation. The retrospective design may introduce bias due to lack of randomization potentially introducing unidentified confounding variables. Specific reasons for transfusion may not have been well documented and non-standardized and thus were not available for review, though univariate results demonstrated increased risk of transfusion with higher energy injuries and more severe fractures. Another limitation is that severity of injury may not have been completely controlled as our analysis was unable to incorporate GA classification into our univariate or multivariate analysis due to incomplete and inconsistent documentation in operative and consultation notes. Therefore, fracture severity may confound the results as preliminary analysis did have more severe injuries in the nonunion cohort as evidenced by higher rates of open injuries and higher GA classification. Additionally, as many of these patients sustained high energy injury mechanisms, the indication for transfusion may have been due to other severe non-orthopedic injuries. Because of the sub-analyses completed, power may have been inadequate to assess outcomes, especially in the closed fracture cohort where each of the outcomes was relatively uncommon. Lastly, nonunion and FRI were non-standardized and determined at the discretion of the treating surgeon for each patient, though the large number of patients, similar practice styles, and fellowship-trained trauma faculty between the two hospital centers should have limited this effect.

## Conclusion

This multicenter study demonstrated that nonunion risk may be associated with peri-operative red blood cell transfusion in open tibial shaft fractures. No independent association was identified for FRI with open or closed fractures. This study adds to the growing literature on the impact of transfusion on outcomes after orthopedic injuries. Surgeons may consider indications for transfusion after open tibia fracture fixation to minimize risk of poor outcomes.

## Supplementary Information

Below is the link to the electronic supplementary material.

Supplementary Material 1

Supplementary Material 2

## Data Availability

No datasets were generated or analysed during the current study.
