# Supplementary material for: Association between perioperative allogenic blood transfusion and risk of fracture related infection and nonunion in operatively treated tibial shaft fractures
Source: Eur J Orthop Surg Traumatol. 2026 Apr 29;36(1):184. doi: 10.1007/s00590-026-04759-1 (PMC13128742; doi:10.1007/s00590-026-04759-1)
Supplement: Supplementary file 1 — Supplementary Material 1 [file 590_2026_4759_MOESM1_ESM.docx]

Supplemental Table 1. Demographic and injury factors associated with fracture related infection (FRI) for sub-analysis group of patients with open fracture. BMI = body mass index.

|  | **Total**  **(N=230)** | **No FRI**  **(N=194)** | **FRI**  **(N=36)** | **p-value** |
| --- | --- | --- | --- | --- |
| Age (years) | 43 ± 17 | 44 ± 18 | 42 ± 15 | 0.6^a^ |
| Male Sex | 160 (70%) | 135 (70%) | 25 (69%) | 0.9^b^ |
| Obese (BMI > 30 kg/m^2^) | 81 (35%) | 67 (35%) | 14 (39%) | 0.6^b^ |
| Current Tobacco Use | 123 (54%) | 100 (52%) | 23 (64%) | 0.2^b^ |
| Diabetes Mellitus | 43 (19%) | 34 (18%) | 9 (25%) | 0.3^b^ |
| High Energy Mechanism | 174 (76%) | 144 (74%) | 30 (83%) | 0.2^b^ |
| Fracture Pattern |  |  |  | 0.9^b^ |
| Simple (AO/OTA 42A) | 84 (37%) | 72 (37%) | 12 (33%) |  |
| Wedge (AO/OTA 42B) | 106 (46%) | 88 (45%) | 18 (50%) |  |
| Complex (AO/OTA 42C) | 40 (17%) | 34 (18%) | 6 (17%) |  |
| Definitive Fixation Method |  |  |  | 0.7^b^ |
| Intramedullary Nail (IMN) | 194 (84%) | 162 (84%) | 32 (89%) |  |
| Open Reduction Internal Fixation (ORIF) | 22 (10%) | 20 (10%) | 2 (6%) |  |
| IMN + Open Plating | 14 (6%) | 12 (6%) | 2 (6%) |  |
| Transfusion | 84 (37%) | 68 (35%) | 16 (44%) | 0.3^b^ |
| Values reported as Mean ± Standard Deviation for continuous variables and N (%) for categorical variables  All P-values considered significant if < 0.050 and are highlighted in bold.  a = Calculated using Student’s T-Test; b = Calculated using Pearson’s Chi-Squared Test | | | | |
