# Supplementary material for: Association between perioperative allogenic blood transfusion and risk of fracture related infection and nonunion in operatively treated tibial shaft fractures
Source: Eur J Orthop Surg Traumatol. 2026 Apr 29;36(1):184. doi: 10.1007/s00590-026-04759-1 (PMC13128742; doi:10.1007/s00590-026-04759-1)
Supplement: Supplementary file 2 — Supplementary Material 2 [file 590_2026_4759_MOESM2_ESM.docx]

Supplemental Table 2. Demographic and injury factors associated with nonunion for sub-analysis group of patients with open fracture. BMI = body mass index.

|  | **Total**  **(N=230)** | **No Nonunion**  **(N=167)** | **Nonunion**  **(N=63)** | **p-value** |
| --- | --- | --- | --- | --- |
| Age (years) | 43 ± 17 | 44 ± 18 | 43 ± 16 | 0.9^a^ |
| Male Sex | 160 (70%) | 110 (66%) | 50 (79%) | **0.04^b^** |
| Obese (BMI > 30 kg/m^2^) | 81 (35%) | 62 (37%) | 19 (30%) | 0.3^b^ |
| Current Tobacco Use | 123 (54%) | 82 (49%) | 41 (65%) | **0.03^b^** |
| Diabetes Mellitus | 43 (19%) | 29 (17%) | 14 (22%) | 0.4^b^ |
| High Energy Mechanism | 174 (76%) | 124 (74%) | 50 (79%) | 0.4^b^ |
| Fracture Pattern |  |  |  | **0.01^b^** |
| Simple (AO/OTA 42A) | 84 (37%) | 71 (43%) | 13 (21%) |  |
| Wedge (AO/OTA 42B) | 106 (46%) | 71 (43%) | 35 (56%) |  |
| Complex (AO/OTA 42C) | 40 (17%) | 25 (15%) | 15 (24%) |  |
| Definitive Fixation Method |  |  |  | 0.9^b^ |
| Intramedullary Nail (IMN) | 194 (84%) | 141 (84%) | 53 (84%) |  |
| Open Reduction Internal Fixation (ORIF) | 22 (10%) | 16 (10%) | 6 (10%) |  |
| IMN + Open Plating | 14 (6%) | 10 (6%) | 4 (6%) |  |
| Transfusion | 84 (37%) | 54 (32%) | 30 (48%) | **0.03^b^** |
| Values reported as Mean ± Standard Deviation for continuous variables and N (%) for categorical variables  All P-values considered significant if < 0.050 and are highlighted in bold.  a = Calculated using Student’s T-Test; b = Calculated using Pearson’s Chi-Squared Test | | | | |
